# Infrared Spectroscopy During Electrocatalytic Turnover Reveals the Ni-L Active Site State During H_2_ Oxidation by a NiFe Hydrogenase**

**DOI:** 10.1002/anie.201502338

**Published:** 2015-04-29

**Authors:** Ricardo Hidalgo, Philip A Ash, Adam J Healy, Kylie A Vincent

**Affiliations:** Department of Chemistry, University of Oxford, Inorganic Chemistry LaboratorySouth Parks Road, Oxford, OX1 3QR (UK)

**Keywords:** biocatalysis, electrochemistry, hydrogenase, in situ spectroscopy, IR spectroscopy

## Abstract

A novel in situ IR spectroscopic approach is demonstrated for the characterization of hydrogenase during catalytic turnover. *E. coli* hydrogenase 1 (Hyd-1) is adsorbed on a high surface-area carbon electrode and subjected to the same electrochemical control and efficient supply of substrate as in protein film electrochemistry during spectral acquisition. The spectra reveal that the active site state known as Ni-L, observed in other NiFe hydrogenases only under illumination or at cryogenic temperatures, can be generated reversibly in the dark at ambient temperature under both turnover and non-turnover conditions. The observation that Ni-L is present at all potentials during turnover under H_2_ suggests that the final steps in the catalytic cycle of H_2_ oxidation by Hyd-1 involve sequential proton and electron transfer via Ni-L. A broadly applicable IR spectroscopic technique is presented for addressing electrode-adsorbed redox enzymes under fast catalytic turnover.

Oxidation and production of H_2_ using earth-abundant metal catalysts is a key challenge for the development of sustainable energy technologies. Hydrogenases, widespread in the microbial world, are finely tuned for these reactions, and use active sites based on iron, or nickel and iron. The fundamental active-site structure for the nickel iron (NiFe) hydrogenases is well-established. The Ni is coordinated by two terminal cysteines, and a further two bridging cysteines which link to Fe. The Fe is additionally coordinated by two CN^−^ and one CO. Fast transport of electrons between physiological donors or acceptors and the active site is achieved via a chain of iron sulfur clusters. Recent work has shown that the potential of the proximal cluster (nearest to the active site) is important in tuning the properties of the active site.[1] Certain O_2_-tolerant NiFe hydrogenases, including *Escherichia coli* hydrogenase 1 (Hyd-1) have an unusual proximal cluster structure,[2] which has been shown by electron paramagnetic resonance (EPR) spectroscopy to undergo two redox transitions at positive potentials.[3]

IR spectroscopy and spectroelectrochemistry has been particularly useful in evaluating active site redox states in NiFe hydrogenases because the intrinsic CO and CN^−^ ligands give rise to vibrational bands (ν_CO_ and ν_CN_) in the mid-IR, the frequency of which is highly sensitive to changes in electron density at the active site.[4] Despite subtle variations between hydrogenases from different organisms and cellular environments a common picture of active site states that are important in catalysis has emerged. These are typically presented as a catalytic cycle containing three states, Ni-SI, Ni-R, and Ni-C (Scheme 1, black solid arrows) on the basis that they have been observed under physiologically relevant conditions.[1a, 4, 5] In this cycle, H_2_ reacts with the EPR-silent Ni-SI state and is cleaved heterolytically[6] to form Ni-R, which is also EPR silent and incorporates a bridging hydride.[7] The initial acceptor for the proton from cleavage of H_2_ remains unclear, although long-range proton transfer channels have been identified.[8] In Scheme 1 we show a generic proton-acceptor, B. Removal of this proton and an electron generates Ni-C, which retains a hydride at the active site.[9] Concerted transfer of a proton and an electron would then be required to regenerate Ni-SI directly from Ni-C ready to restart the catalytic cycle. Alternative pathways for conversion of Ni-C to Ni-SI have been proposed on the basis of DFT calculations by Hall and co-workers, in which the proton- and electron-transfer steps are separated,[10] and in the first cycle of an autocatalytic mechanism.[11] In these pathways an additional intermediate is required in which the bridging hydrogen is transferred to a nearby base as a proton, resulting in a formally Ni^I^Fe^II^ state of the active site. Sequential proton- and electron-transfer steps via a Ni^I^ intermediate are well-established for small molecule H_2_-production catalysts based on Ni.[12]

**Scheme 1:**
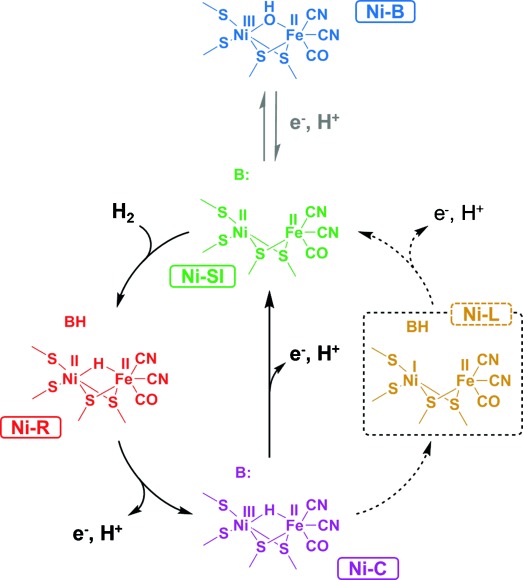
H_2_ oxidation (black) and anaerobic inactivation (gray) of NiFe hydrogenase.

An active site state at the Ni^I^Fe^II^ redox level has been identified for most NiFe hydrogenases following photo-illumination of Ni-C at cryogenic temperatures.[4] This state, termed Ni-L, is characterized by unusually low wavenumber ν_CO_ and ν_CN_ bands. It has been suggested by Neese and co-workers that the additional electron density resides in a metal–metal bond rather than being localized on Ni, with a nearby thiolate base suggested as the proton acceptor.[13] Protonation of one of the cysteinyl ligands to the active site in the Ni-L state has been observed by Siebert et al.[14] The specialized conditions required for Ni-L formation mean that this state has generally been assumed to be irrelevant to the catalytic cycle. A previous report of Ni-L at ambient temperature attributed its appearance to a damaged, inactive, form of the enzyme.[15] Interestingly, Rauchfuss and co-workers demonstrated a Ni^I^-containing bimetallic model for Ni-L which is reduced by H_2_ to give a species containing a bridging hydride.[16]

Direct experimental evidence for which states of the NiFe active site are involved in the catalytic mechanism is limited. The majority of spectroelectrochemical studies have used hydrogenase in solution either with[5, 17] or without[18] redox mediators: owing to the very high turnover frequency of NiFe hydrogenases (>10 000 s^−1^),[19] even studies carried out in H_2_-saturated static solutions are likely to suffer from substrate depletion and limited electron delivery.[20] The approach of protein film electrochemistry (PFE) addresses this, allowing rapid, direct electron transfer between the enzyme and an electrode with efficient mass transport provided by electrode rotation so that the magnitude of the current reports on enzyme activity at each potential (Figure 1 a).[20, 21] A technique known as surface enhanced IR absorption (SEIRA) spectroscopy has been widely used to study redox enzymes immobilized on gold electrodes,[22] notably activation of the membrane bound NiFe hydrogenase from *Ralstonia eutropha* in the presence and absence of H_2_,[23] and complex I from *E. coli*.[24] Herein we describe a new approach to combining the electrochemical control of PFE with structural insight provided by IR spectroscopy, a technique we term protein film infrared electrochemistry (PFIRE; Figure 1 b; Supporting Information, Figure S1). The enzyme, Hyd-1, is adsorbed on a high surface area carbon electrode constructed from carbon black deposited onto a Si internal reflection element (IRE).[25] Flow of H_2_-saturated electrolyte through a custom-built spectroelectrochemical ATR-IR cell provides efficient mass transport, shown by the similar waveshape of the catalytic voltammogram recorded in this cell (Figure 1 c) with that of Hyd-1 on a planar rotating disc electrode (RDE, Figure 1 a). The higher current in the ATR-IR cell reflects the larger sample of enzyme under electrochemical control on the high surface area electrode. Figure 1 d shows the ν_CO_ and ν_CN_ region of an IR spectrum for Hyd-1 recorded in this cell, with the active site in the oxidized, catalytically inactive state, known as Ni-B, formed from Ni-SI at high potential (Scheme 1).

**Figure 1:**
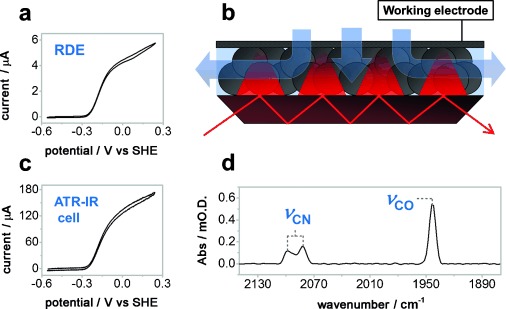
Voltammogram of Hyd-1 recorded at 10 mV s^−1^, pH 6.0, and 1 bar H_2_ on a RDE (2000 rpm); b) diagram of the working-electrode configuration and solution flow (blue arrows) over the multi-bounce Si IRE; c) Voltammogram in the spectroelectrochemical ATR-IR cell described herein under the same conditions as (a); d) IR spectrum of the most oxidized state of Hyd-1, Ni-B, recorded under Ar in the ATR-IR cell.

Figure 2 shows spectra of the ν_CO_ region recorded at selected electrode potentials, under 1 bar Ar or H_2_, on a single sample of adsorbed Hyd-1, together with the corresponding current–time trace. The adsorbed enzyme was first activated at low potential under H_2_. At the lowest potential applied, −0.594 V, there is no detectable electrocatalytic current under either Ar or H_2_, consistent with the poor ability of Hyd-1 to reduce protons, even at pH 6 (Figure 2 a, gray).[26] Under a H_2_ atmosphere, the current increases in magnitude as the electrochemical driving force is raised (Figure 2 a, black). The electrocatalytic current is extremely stable at −0.199 V and −0.074 V over >1000 s, indicating that the high flow rate of electrolyte solution through the cell (62 mL min^−1^) provides effective mass transport of H_2_, and that the enzyme is adsorbed robustly on the carbon. At flow rates greater than 50 mL min^−1^, increasing the flow rate led to no further increase in the electrocatalytic current. At the most positive potential applied (+0.356 V) the current under H_2_ decays over time, consistent with the well-established slow anaerobic, oxidative inactivation of NiFe hydrogenases (Scheme 1).

**Figure 2:**
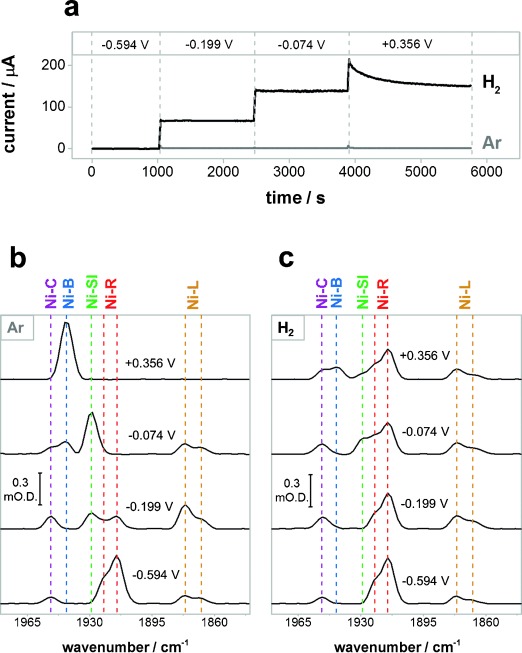
a) Current–time traces of Hyd-1 in the ATR-IR cell in Ar-saturated (gray) and H_2_-saturated (black) buffer; b), c) spectra showing the ν_CO_ region at each potential under Ar (b) and H_2_ (c). Potentials are quoted in volts (V) relative to the standard hydrogen electrode (SHE).

The ν_CO_ and ν_CN_ bands observed in spectra at different potentials under Ar (Figure 2 b; Supporting Information, Figure S2) reveal a set of active site states for Hyd-1 consistent with states observed in solution IR spectra of other NiFe hydrogenases (Supporting Information, Table S1).[15, 17a, 27] At the most negative potential, the dominant state is Ni-R. Two forms of Ni-R are present, indicated by two ν_CO_ bands that are always present in the same ratio of intensities; for other hydrogenases these have been attributed to forms of Ni-R with different protonation states in the region of the active site.[28] As the potential is stepped to −0.199 V, the population of Ni R decreases, and there is an increase of three species: Ni-C, Ni-SI, and Ni-L. Importantly, these spectra were recorded in the dark, and represent the first observation of Ni-L states in the absence of UV/Vis light and at room temperature. There are also two forms of Ni-L, suggesting several protonation sites. The concentrations of Ni-C and the Ni-L states remain in the same ratio at all potentials, showing that these states are in chemical equilibrium. The concentrations of Ni-L, Ni-C, and Ni-R decrease as the potential is stepped up to −0.074 V and Ni-SI is the dominant state at this potential. At the most positive applied potential (+0.356 V), Ni-B is the only state detected, showing that the whole enzyme sample has responded to application of potential. A more detailed redox characterization of the active site states of Hyd-1 under N_2_ (Supporting Information, Figure S3) shows that these changes are fully reversible with potential. The adsorbed enzyme sample is very stable: after 48 h of experiments at 20 °C there is no detectable change in the ν_CO_ intensity for the active site (Supporting Information, Figure S4).

Figure 2 c shows spectra recorded at the same potentials, but under 1 bar H_2_ (ca. 0.8 mm in solution). At −0.594 V, a potential at which there is no detectable catalytic turnover, there are no obvious differences to the spectrum under Ar. However at more positive potentials where Hyd-1 engages in electrocatalysis under a H_2_ atmosphere, there are significant differences in the distribution of states. Under H_2_ Ni-R persists as the majority species at all applied potentials, together with a significant proportion of Ni-C and Ni-L, which are now observed even at +0.356 V where they are absent under an inert atmosphere. Under H_2_, Ni-SI is observed in a lower concentration at −0.074 V and persists up to the highest potential, and the onset potential for formation of Ni-B is more positive.

The differences between the Ar and H_2_ data can be explained by reference to a catalytic cycle involving the Ni-SI, Ni-R, Ni-C, and Ni-L states (Scheme 1). At intermediate potentials (−0.199 and −0.074 V), the greater concentration of Ni-R and lower concentration of Ni-SI under H_2_ is consistent with fast attack on Ni-SI by substrate: the H_2_ concentration is well above the Michaelis Menten constant for Hyd-1 (9 μm).[29] At the highest potential (+0.356 V), the concentration of Ni-B increases and the concentration of Ni-SI decreases with time, consistent with high potential inactivation shown by the decay in current (Figure 2 a; Supporting Information, Figure S5). Observation of Ni-L and Ni-C under H_2_ at the highest potential, outside the potential window where they are observed under non-turnover conditions (Supporting Information, Figure S3), provides strong evidence that both Ni-C and Ni-L are generated in response to catalytic H_2_ oxidation. The concentrations of Ni-C and Ni-L remain in the same ratio at all potentials, as observed under Ar, supporting a potential-independent equilibrium between these two states. Under conditions of catalytic H_2_ oxidation, the total concentration of Ni-C and Ni-L remains constant. This suggests that the rate of formation of Ni-C from Ni-R is similar to the rate of consumption of Ni-L to form Ni-SI, and that the rate of both electron transfer steps in Scheme 1 have the same potential dependence.

Recent EPR measurements at cryogenic temperatures have shown the presence of Ni-L in Hyd-1 without external illumination,[3] and Ni-L conversion to Ni-SI in *Desulfovibrio vulgaris* Miyazaki F hydrogenase has been reported by Tai et al.,[30] who suggest that formation of Ni-L requires the proximal cluster to be reduced. Our observation of Ni-L in Hyd-1 at intermediate potentials under an inert atmosphere is consistent with these reports since the proximal cluster is known to be in the fully-reduced 3+ state at these potentials.[3] Furthermore, our demonstration that Ni-L is formed reversibly and in response to catalytic H_2_ oxidation at room temperature in the dark provides strong evidence that it is the intermediate in conversion between Ni-C and Ni-SI in the catalytic cycle of Hyd-1.

In summary, this study builds upon the direct electrochemical control provided by the technique of PFE which has been important in addressing the activity and inhibition of redox enzymes via variations in current under a range of solution conditions. We have demonstrated a method for coupling PFE, which provides no direct structural information, with IR spectroscopy at a carbon electrode to provide insight into the active site states present during catalytic H_2_ oxidation by a NiFe hydrogenase. The high surface area carbon electrode used in this work provides facile, robust adsorption of the enzyme without the need for specific surface modification. This new approach adds structural insight to the information available from electrochemistry alone and will be widely applicable to redox proteins involved in small molecule binding and activation.

## Supporting Information

As a service to our authors and readers, this journal provides supporting information supplied by the authors. Such materials are peer reviewed and may be re-organized for online delivery, but are not copy-edited or typeset. Technical support issues arising from supporting information (other than missing files) should be addressed to the authors.

miscellaneous_information
