# Supplementary material for: Infrared Spectroscopy During Electrocatalytic Turnover Reveals the Ni-L Active Site State During H2 Oxidation by a NiFe Hydrogenase
Source: Angew Chem Int Ed Engl. 2015 Apr 29;54(24):7110–3. doi: 10.1002/anie.201502338 (PMC4531817; doi:10.1002/anie.201502338)
Supplement: Supplementary file 1 — miscellaneous_information [file anie0054-7110-sd1.pdf]

Angewandte Chemie
Eine Zeitschrift der Gesellschaft Deutscher Chemiker

Supporting Information

German Edition: DOI:

Infrared Spectroscopy During Electrocatalytic Turnover Reveals the Ni-L Active Site State During H_2 Oxidation by a NiFe Hydrogenase**

*Ricardo Hidalgo, Philip A. Ash, Adam J. Healy, and Kylie A. Vincent**

anie_201502338_sm_miscellaneous_information.pdf

Supporting Information

Experimental Methods

Sample preparation:

Escherichia coli hydrogenase 1 (Hyd-1) was prepared according to a published procedure and concentrated to 6.3 mg mL^{-1}.[1] A mixed buffer solution (pH 6.0) was prepared according to a published procedure and contained NaCl (Sigma, 100 mM) as supporting electrolyte. Carbon black particles (Black Pearls 2000, Cabot Corporation, used as received) were dispersed in water by sonication to a loading of 20 mg mL^{-1}. A 50 μL aliquot of Hyd-1 was mixed with 5 μL of the carbon black dispersion and this mixture was left at 0 ^{o}C for 2 h to allow adsorption of enzyme. The enzyme-modified particles were then washed by centrifugation to remove any unadsorbed Hyd-1 and concentrated as necessary to maintain a carbon black loading of 20 mg mL^{-1}. Ultra high purity water (MilliQ Millipore, 18 MΩ cm) was used throughout. All enzyme adsorption was carried out in a N_2-filled glove box (<1 ppm O_2, Glove Box Technology Ltd), connected by purged antechambers to a second glove box which was maintained under a dry N_2 atmosphere (< 85 °C dew point) for spectroscopic measurements.

Protein film infrared electrochemistry (PFIRE) measurements:

Infrared spectroscopic measurements were carried out using the external beam of a Varian 680-IR spectrometer, controlled by Resolutions Pro software. The spectrometer was placed on a table connected to the dry glove box and the IR beam was diverted into the glove box through a short purged tube and 5 mm thick KBr window. Inside the glovebox the IR beam was diverted onto an off-axis parabolic focussing mirror and into a multiple-reflection ATR accessory (PIKE Technologies, custom-modified GladiATR). A trapezoidal Si internal reflection element (IRE, Crystal Gmbh, 8.39 × 5 × 1 mm^3) with a face angle of 39° was sealed into a polyether ether ketone (PEEK) baseplate using silicone sealant and the spectroelectrochemical cell (described below) was sealed onto the baseplate. After exiting the ATR accessory the IR beam was detected by a narrow-band mercury cadmium telluride detector cooled to 77 K. The states of the NiFe active site observed were identical in spectra recorded both with and without optical filters: low-pass (Agilent, <3950 cm^{-1}); wide band pass (Northumbria Optical Coatings, 4.75 – 5.66 μm).

Spectral processing was carried out using Origin 9.1. A baseline was subtracted from the data using an interpolated spline function; care was taken to avoid distorting peak shapes through the choice of baseline anchors with reference to corresponding 2nd derivative spectra. Peaks in the ν_{CO} region of baseline-subtracted spectra were fitted to Gaussian profiles, and the fitted peak heights used to plot the titrations shown in Figure S2. The central wavenumber of each state of the Hyd-1 active site was found to be independent of potential, and these are reported in Table S1.

ATR-IR spectroelectrochemical cell for PFIRE measurements:

A small volume spectroelectrochemical cell was constructed from PEEK, with a total volume of *ca.* 250 μL. The cell body houses the electrodes and electrode connections: a miniature saturated

calomel reference electrode (SCE, constructed as described below); a Pt wire counter electrode (Surepure Chemetals, 99.99 %, 26 gauge); and a carbon rod (WH Smith, 0.9 mm HB) working electrode connection, which allows electrical connection to a high surface area carbon black working electrode, shown schematically in Figure S1. To form the working electrode 1 μL of the carbon black particles modified with Hyd-1 was drop cast to completely cover the Si IRE and allowed to partially dry to give a working electrode area of approximately 8.39 × 5 mm^2 and a thickness of *ca* 1 μm. A piece of carbon paper (Toray, TGP-H-030), commonly used as a gas diffusion layer in fuel cell applications, was placed on top of the carbon black electrode to provide good lateral connectivity. An additional inlet and outlet provide solution access to the cell allowing N_2-, Ar- or H_2-saturated buffer to flow through the cell *via* a peristaltic pump (QL-1000, Williamson Pumps Ltd). Gas access to the buffer is provided by a set of mass flow controllers (Brooks) and all gases used were passed through an O_2-removal filter (Restek Super-Clean, gas outlet quality >99.9999%). The Si IRE was cleaned by successive ultrasonication in sulphuric acid, piranha solution and water. Electrochemical control was provided by an Autolab PGSTAT 128N. Prior to experiments Hyd-1 was activated under 1 bar H_2 at -0.594 V for 1 hour. Potentials (*E*) quoted in the text are adjusted to volts (V) *vs* the standard hydrogen electrode (SHE) using the conversion *E* (V vs SHE) = *E* (V vs SCE) + 0.241 V at 25 °C.

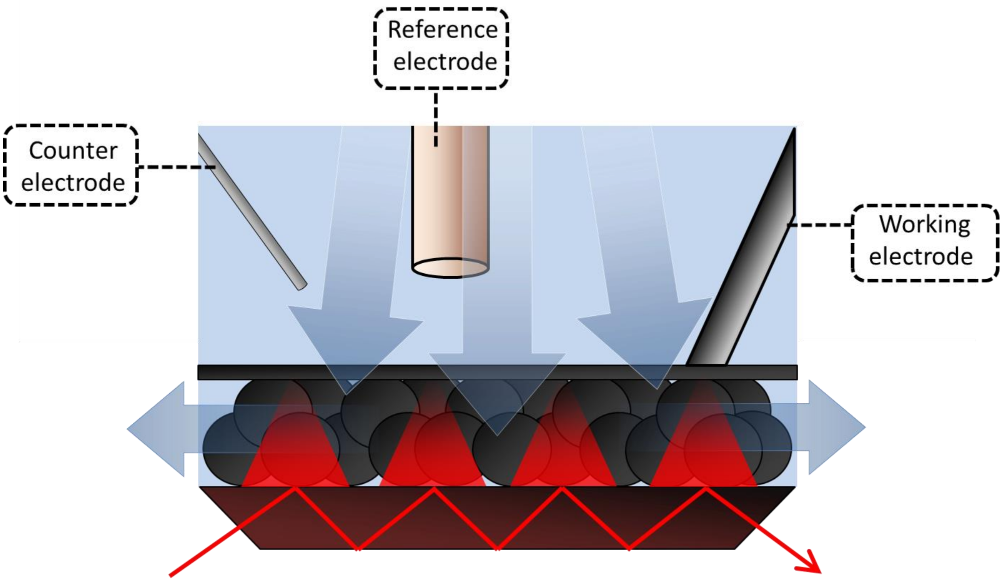

Figure S1. Schematic representation of the spectroelectrochemical ATR-IR cell designed for PFIRE experiments, showing the relative location of electrodes and the direction of solution flow. The enzyme, Hyd-1, is adsorbed on carbon beads which are cast directly onto the Si internal reflection element.

Miniature saturated calomel electrode (SCE):

A 5 μL aliquot of mercury and *ca* 30 mg of mercury(I) chloride were added to a glass fritted plastic tube. An electrical connection was made to the mercury pool *via* a platinum wire. This plastic tube was then fitted into a 5 cm long, 2 mm diameter leak-free PEEK reference electrode body (Harvard Apparatus UK) and both sections were filled with saturated KCl solution. This home-built miniature SCE provided very stable potentials over long periods of time (less than 5 mV variation in >1 month).

Supporting Data

Figure S2. Infrared spectra of *E.coli* Hyd-1 in the PFIRE cell, poised at different potentials under an Ar atmosphere. These are the same spectra as reported in Figure 2 of the main text but over an expanded wavenumber range to show the ν_{CN} as well as the ν_{CO} region.

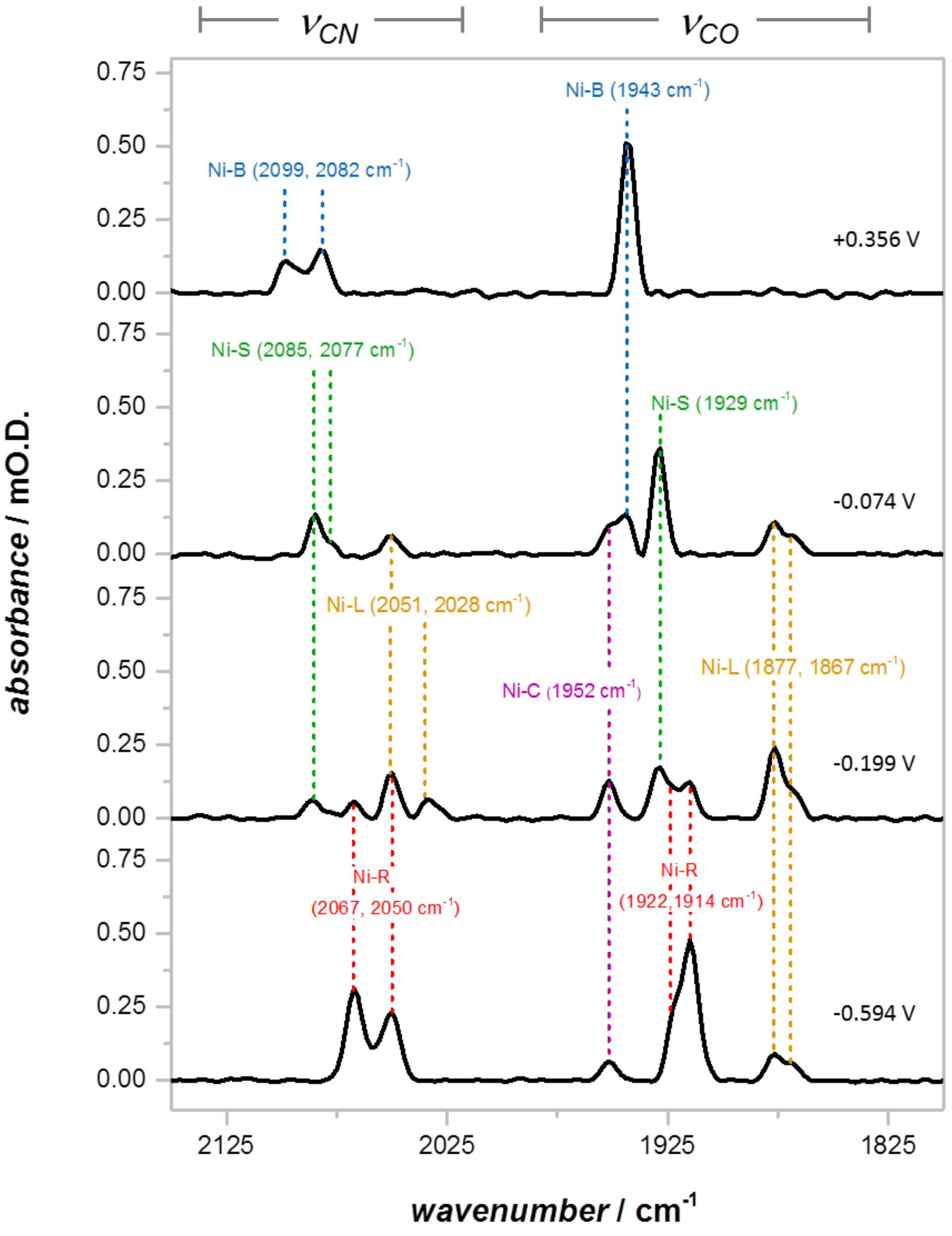

Figure S3. Plot of normalized ν_{CO} peak intensities for (a) oxidative and (b) reductive potential titrations of *E.coli* Hyd-1 immobilised on a carbon black particle electrode at pH 6.0 under N_2. Lines show Nernstian fits for *n*=1 processes (*n*=number of electrons transferred).

a

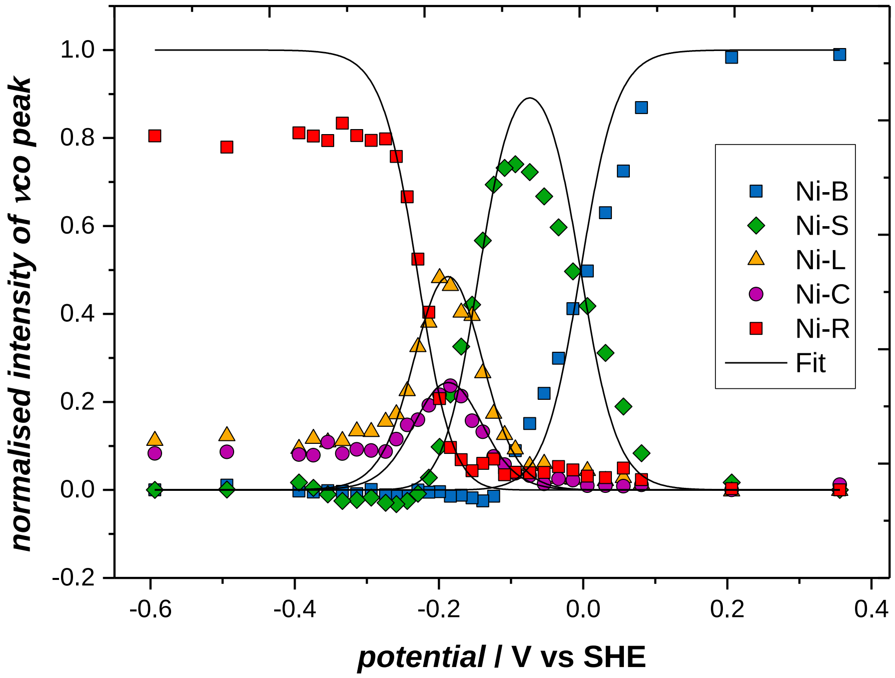

b

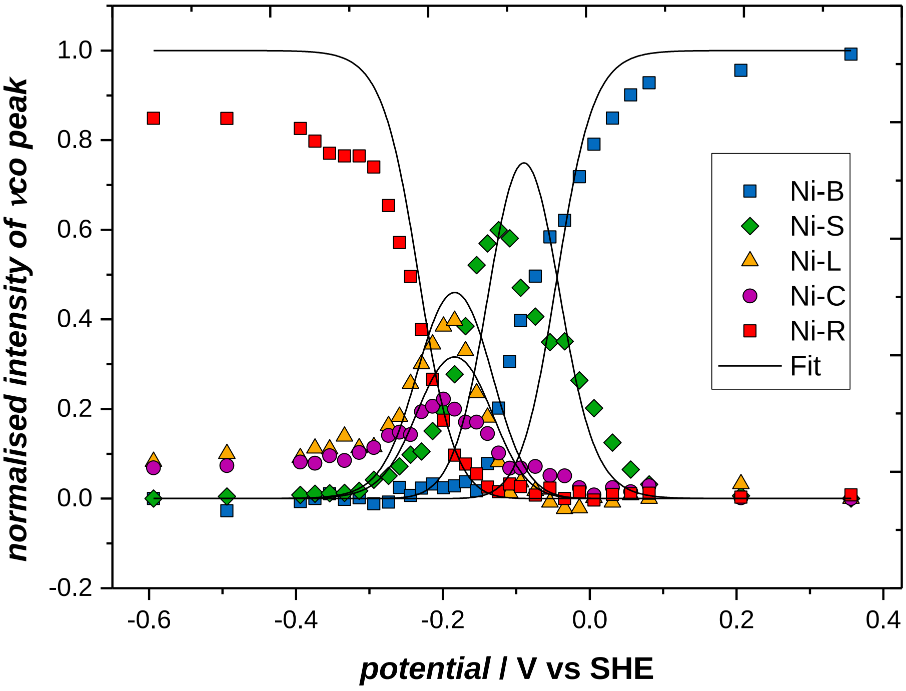

Table S1. Comparison of central wavenumber of ν_{CO} and ν_{CN} peaks of the different redox states for several hydrogenases. The underlined values were recorded at low temperature upon illumination of Ni-C.

**redox state**	***E.coli*** **Hyd-1 pH 6.0**		***Aquifex aeolicus*** **membrane bound hydrogenase I[2] pH 6.4**		***Ralstonia eutropha*** **membrane bound hydrogenase[3] pH 5.5**		***Allochromatium vinosum*** **hydrogenase pH 6.0 [9.0][4]**	
	ν_{CO}	ν_{CN}	ν_{CO}	ν_{CN}	ν_{CO}	ν_{CN}	ν_{CO}	ν_{CN}
**Ni-A**	-	-	-	-	-	-	1945	2082<br>2093
**Ni-B**	1943	2082<br>2099	1939	2081<br>2092	1948	2081<br>2098	1943	2079<br>2090
**Ni-SU**	-	-	-	-	1943	2082<br>2104	1948	2088<br>2100
**Ni-SI$_{I}$**	-	-	-	-	1910	2055<br>2063	1910	2052<br>2067
**Ni-SI$_{II}$**	1929	2085<br>2077	1927	2077<br>2087	1936	2075<br>2093	1931	2073<br>2084
**Ni-C**	1952	-	1949	2078<br>2088	1957	2075<br>2097	1951	2073<br>2085
**Ni-L$_{II}$**	-	-	1900	2049<br>2068	1899	2040<br>2065	[1898]	[2044]<br>[2059]
**Ni-L$_{III}$**	1877	2028<br>2051	1876	2033<br>2057	-	-	-	-
**Ni-L$_{I}$**	1867	-	1862	2024<br>2045	-	-	-	-
**Ni-R$_{I}$**	-	-	-	-	1948	2068<br>2087	1936	2059<br>2072
**Ni-R$_{II}$**	1914	2050<br>2067	1910	2047<br>2066	1926	2049<br>2075	1921	2048<br>2064
**Ni-R$_{III}$**	1922		-	-	1919	2046<br>2071	1913	2043<br>2058

Figure S4. Spectra showing the ν_{CO} peak of the most oxidized state, Ni-B, of Hyd-1. Black: At the beginning of the reductive titration and red: at the end of the oxidative titration approximately 48 hours later. The potential for both measurements was +0.115 V vs SHE.

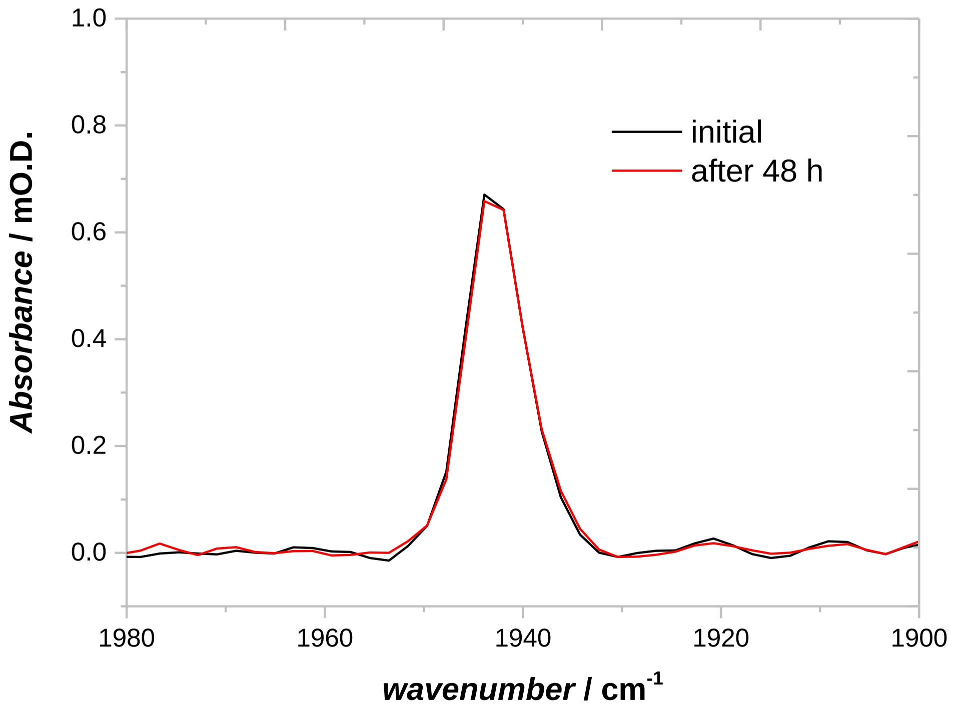

Figure S5. Inactivation of Hyd-1 *via* the formation of Ni-B from Ni-SI. (a) Current-time trace for the more positive potential steps under H_2. Panel b shows spectra recorded during each of the gray shaded time periods indicated in the current-time trace. The spectrum b_i is an absolute spectrum indicating the species present at the beginning of the potential step. Spectrum b_{ii}, recorded at a later time at the same potential and reported as a difference spectrum relative to b_i, shows that there is no change in the distribution of active site states during catalytic H_2 oxidation, consistent with the stability of the current. Spectra b_{iii} and b_{iv}, also reported as difference spectra relative to b_i, show the conversion of Ni-SI to Ni-B during high potential inactivation.

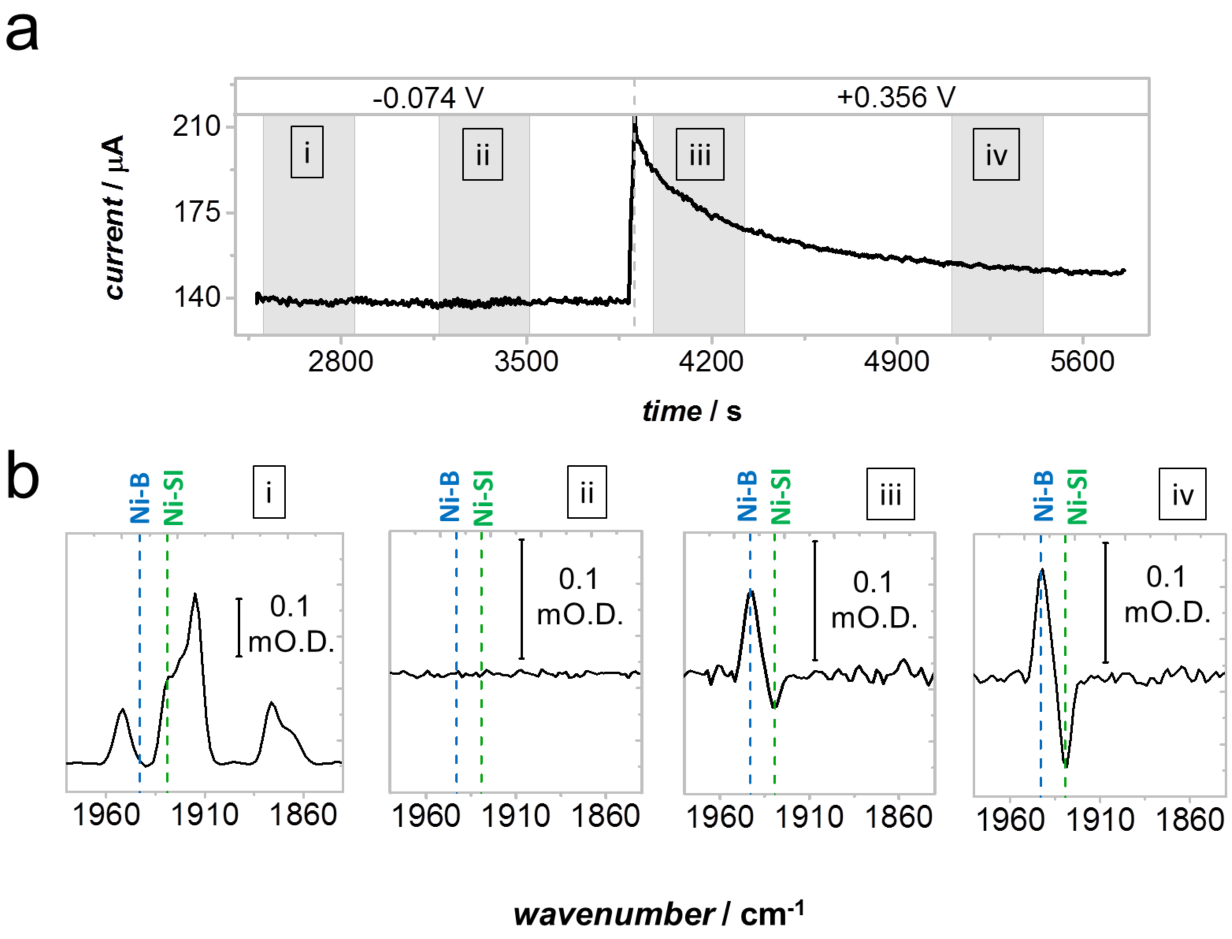

Supporting References

[1] M. J. Lukey, A. Parkin, M. M. Roessler, B. J. Murphy, J. Harmer, T. Palmer, F. Sargent, F. a. Armstrong, *J. Biol. Chem.* **2010**, *285*, 3928-3938.

[2] a) M.-E. Pandelia, P. Infossi, M. Stein, M.-T. Giudici-Orticoni, W. Lubitz, *Chem. Commun.* **2012**, *48*, 823-825; b) M.-E. Pandelia, V. Fourmond, P. Tron-Infossi, E. Lojou, P. Bertrand, C. Léger, M.-T. Giudici-Orticoni, W. Lubitz, *J. Am. Chem. Soc.* **2010**, *132*, 6991-7004.

[3] a) A. J. Pierik, M. Schmelz, O. Lenz, B. Friedrich, S. P. Albracht, *FEBS Lett.* **1998**, *438*, 231-235; b) M. Saggu, I. Zebger, M. Ludwig, O. Lenz, B. Friedrich, P. Hildebrandt, F. Lendzian, *J. Biol. Chem.* **2009**, *284*, 16264-16276.

[4] a) K. A. Bagley, E. C. Duin, W. Roseboom, S. P. J. Albracht, W. H. Woodruff, *Biochemistry* **1995**, *34*, 5527-5535; b) B. Bleijlevens, F. A. van Broekhuizen, A. L. De Lacey, W. Roseboom, V. M. Fernandez, S. P. J. Albracht, *J. Biol. Inorg. Chem.* **2004**, *9*, 743-752.
